# A Smartphone App for Attentional Bias Retraining in Smokers: Mixed Methods Pilot Study

**DOI:** 10.2196/22582

**Published:** 2022-01-03

**Authors:** Carol C Choo, Yi Zhuang Tan, Melvyn W B Zhang

**Affiliations:** 1 College of Healthcare Sciences, Division of Tropical Health and Medicine, James Cook University Queensland Australia; 2 Department of Psychology, James Cook University Singapore Singapore; 3 Family Medicine and Primary Care, Lee Kong Chian School of Medicine, Nanyang Technological University Singapore Singapore

**Keywords:** attentional bias retraining, smartphone app, mixed methods, smoking, mobile phone

## Abstract

**Background:**

Smoking is a global health threat. Attentional bias influences smoking behaviors. Although attentional bias retraining has shown benefits and recent advances in technology suggest that attentional bias retraining can be delivered via smartphone apps, there is a paucity of research on this topic.

**Objective:**

This study aims to address this gap by exploring the use of attentional bias retraining via a novel smartphone app using a mixed methods pilot study. In the quantitative phase, it is hypothesized that participants in the training group who undertake attentional bias retraining via the app should have decreased levels of attentional bias, subjective craving, and smoking frequency, compared with those in the control group who do not undertake attentional bias retraining. The qualitative phase explores how the participants perceive and experience the novel app.

**Methods:**

In all, 10 adult smokers (3 females and 7 males) between the ages of 26 and 56 years (mean 34.4 years, SD 9.97 years) were recruited. The participants were randomly allocated to the training and control groups. In weeks 1 and 3, participants from both groups attempted the standard visual probe task and rated their smoking frequency and subjective craving. In week 2, the participants in the training group attempted the modified visual probe task. After week 3, participants from both groups were interviewed about their views and experiences of the novel app.

**Results:**

The results of the quantitative analysis did not support this study’s hypothesis. The qualitative data were analyzed using thematic analysis. The results yielded 5 themes: ease, helpfulness, unhelpful aspects, barriers, and refinement.

**Conclusions:**

Findings from the qualitative study were consistent with those from previous studies on health-related smartphone apps. The qualitative results were helpful in understanding the user perspectives and experiences of the novel app, indicating that future research in this innovative area is necessary.

## Introduction

### Background

Smoking remains a global health threat [1,2], and it is compounded by adverse relationships with depression and anxiety [3], as well as the consequences of secondhand smoke [4]. During the COVID-19 pandemic, the issue of tobacco smoking has become salient, as there is increasing evidence suggesting that smoking is associated with COVID-19 severity [1]. Despite the growing impetus for smoking cessation efforts [1,2], as smoking is prevalent, smokers are constantly exposed to smoking-related cues in the environment [2,5]. Hyperattention to such stimuli imposes obstacles to any attempts at abstinence owing to the unconscious process of attentional bias [5,6]. Theories of attentional bias underscore the relationship between attentional bias and subjective craving, which is a potent predictor of smoking relapse. Attentional bias perpetuates the cycle of addiction, and smokers who exhibit greater attentional bias are more likely to relapse [7]. Incentive sensitization produces attentional processing bias toward substance-related cues because of increased saliency and exacerbates craving toward the substance [8]. In addition, there is a bidirectional relationship between attentional bias cues and subjective craving for a substance [9]. Through classical conditioning, substance-related stimuli become more salient, which increases subjective craving [10]. Consequently, attentional bias toward the substance is further increased, which consolidates the cycle of craving and attentional bias in addiction.

The elaborated intrusion theory of desires makes similar predictions about the reciprocal relationship between attentional bias and subjective craving [11]. Cognitive elaboration on the substance perpetuates craving, which increases the likelihood of consumption. Subjective craving can initially be experienced as an intrusive thought, triggered by external substance-related cues [12], which drives cognitive elaboration. This increases attentional allocation to substance-related stimuli, which, in turn, strengthens craving. The theory of current concerns also supports the relationship between attentional bias toward cue-related substances and subjective craving [13]. Overall, although various theories suggest different mechanisms by which attentional bias develops, they converge on the idea that attentional bias and subjective craving have a bidirectional causal relationship with each other.

Common conceptual frameworks and measures to investigate attentional bias in substance use include the modified version of the Stroop task and the visual probe task. The modified version of the Stroop task in studies of substance builds upon the classical Stroop task [14-16]. Several studies using the Stroop task elicited evidence of smokers’ attentional bias toward smoking-related stimuli [17,18]. These studies found that smoking-related stimuli interfered with smokers’ performance in the Stroop task, suggesting that attention was drawn to such stimuli, thus impairing their performance on the specified tasks [19]. Although the Stroop task is a well-established attentional measure, alternative mechanisms may also explain the impaired performance. When modified for addiction studies, caution must be exercised in drawing the same conclusion on attentional bias underlying the results [20-24]. Another common measure involves the visual probe task [25], which uses images related to smoking and neutral images. Related studies expectedly yielded results showing that smokers displayed attentional bias [26]. The visual probe task has clear advantages over the Stroop task as a measure of attentional bias toward smoking-related stimuli. First, the visual probe task minimizes any response bias because of the motivational state of the participant [25]. Second, it more accurately reflects the real-life scenarios that a smoker faces. The visual probe task requires the participants to split attention between 2 different stimuli. In the real world, smokers constantly have to split their attention between different stimuli; thus, the visual probe task can better capture this aspect, which allows its results to be more generalizable.

### Attentional Bias Retraining in Smoking

Despite advancements in experimental psychology, the field of attentional bias retraining in smoking is relatively new. Emerging research indicates that attentional bias retraining holds potential clinical utility as an adjunct tool to complement smoking interventions. Both theoretical and empirical evidence supports attentional bias retraining. Theories of attentional bias have indicated that attentional bias results from the repeated pairing of cues, such as the sight of cigarettes, which leads to sensitized reactions to such cues, and thus they become more salient. Their saliency perpetuates the vicious cycle as it increases attention to smoking cues, which are related to smoking cessation outcomes [7]. In contrast to smokers who have increased attentional bias toward smoking-related cues, former smokers show avoidance of such cues [27]. Thus, attentional bias retraining in smokers may be crucial for successful smoking cessation.

The first attempt at attentional bias retraining in smokers used a modified version of the visual probe task [28]. This modified version used the same conceptual framework as the original visual probe task, with the only difference being that the dot probe always replaced the neutral stimuli. The results revealed a significant decrease in posttraining attentional bias toward smoking-related stimuli compared with that before training, suggesting that attentional bias retraining can alter such bias. However, subsequent studies using single-session training on smokers were unable to replicate the results [29,30]. Conversely, it was found that multiple sessions of attentional bias retraining produced robust changes in attentional bias toward smoking-related stimuli [31]. However, the challenge lies in convincing the participants to commit to multiple sessions because of the inconvenience these sessions bring to their personal schedules. An efficient way to curtail this difficulty would be to conduct the sessions over mobile devices, given their benefits in high-dosage treatment delivery, prevalent use, and convenience. With the rising trend in e-technology [32-34], the advancement of smartphones has ushered in an era where smartphone apps can be used to enhance the delivery of interventions. The use of mobile devices is widespread worldwide, and this ubiquity enables people with no access to mental health services to have opportunities to seek early treatment with convenience [35,36].

Currently, there is a dearth of research on attentional bias retraining for smokers via mobile devices. We have conducted a literature review and found a paucity of research in the area [37], indicating that research on this topic is pertinent. In the first study of its kind, Kerst et al [38] used PDAs to deliver attentional bias retraining to smokers. The results showed that attentional bias toward smoking-related stimuli and subjective craving decreased over the week in the attentional bias retraining group. However, the generalizability of the results was limited by the sample group, as a large proportion of the sample were African American. Recent research [39,40] has cautioned against assumptions that outcomes from studies conducted in Western countries could be generalized to the native Asian population, as the implementation of any effective intervention should consider the local sociocultural context. Despite this, the study is the first of its kind to report the use of attentional bias retraining intervention via mobile devices and holds promise that warrants further exploration in the local context.

### Objectives

The literature review indicated the absence of studies exploring the use of smartphone apps in retraining attentional bias in smokers [37]. This study aims to address this gap in the literature. As recommended by previous research [41], the employment of a mixed methods study design will be useful in exploring the impact of the novel smartphone app. A mixed methods approach in this study entails the use of both quantitative and qualitative methods [42], with the overall objective of exploring the perspectives and experiences of the novel smartphone app, as well as to evaluate the outcomes of attentional bias retraining. Specifically, although the understanding of user perspectives and experiences of the novel app would be explored using qualitative methodology, the specific outcomes of attentional bias, craving, and smoking frequency would be evaluated using quantitative methodology.

Similar to previous research [41], in this pilot study, we investigated attentional bias retraining delivered via the novel smartphone app by quantitatively analyzing the attentional bias to smoking cues, subjective craving of smoking, and smoking frequency of current adult smokers who did and did not undertake attentional bias retraining. It was hypothesized that participants who did and did not undertake attentional bias retraining via the novel smartphone app would differ in their attentional bias, subjective craving, and smoking frequency. Specifically, participants in the training group who underwent attentional bias retraining would have decreased attentional bias, subjective craving, and smoking frequency when compared with participants in the control group (who did not undertake attentional bias retraining). The qualitative study would be exploratory in nature with the aim of exploring the perceptions and experiences of all the participants who had used the novel app. The research question for the qualitative study was as follows: “How do participants perceive and experience the novel smartphone app?”

## Methods

### Participant Recruitment

The participants were recruited through snowball sampling and by word of mouth. The inclusion criteria were as follows: at the time of the study, the participants should be adult smokers who had been smoking for the past 2 years and they should have access to mobile phones using the Android (Google, Inc) operating system. The exclusion criterion was that the participants were regular users of tobacco products other than cigarettes (eg, electronic cigarettes and waterpipes). In total, 10 participants (3 females and 7 males) completed the study, and their ages ranged from 26 to 56 years (mean 34.4 years, SD 9.97 years). There were 4 Chinese, 4 Malay, and 2 Indian participants. As this was the first study of its kind on a novel smartphone smoking app in the local context, the sample size was kept small, similar to a previous local-level study using a mixed methods design [41].

The visual analog scale was used to measure subjective craving. It consisted of a sliding response from 1 to 10 (1=no craving and 10=extreme craving) to the question “How much are you craving to smoke a cigarette right now?” Higher scores indicated higher subjective cravings. The numerical response to the question “How many cigarettes did you smoke daily for the past week?” was used to measure the smoking frequency. Higher scores indicated a higher smoking frequency.

### App Development

The novel smartphone app was developed on the Android platform in collaboration with our industry partner and coauthor (MWBZ). To the best of our knowledge, this is the only smartphone app that has been developed to retrain attentional bias in smokers. The app used for this study was developed using the research platform by Zhang et al [43] for substance use disorders. The app was developed by our coauthor (MWBZ), along with a freelance developer, using Unity 3D (Unity Technologies Inc), and it was programmed to be compatible with the Android platform. Within the app, participants could undertake either an attention bias assessment task or a bias modification task. The stimulus images used were similar to those used in previous studies [44]. Pictures of smoking-related stimuli and correspondingly matched neutral stimuli were acquired from Woud et al [44], and permission was obtained to embed them in our app. The smartphone app contained a standard visual probe task (assessment task) and a modified visual probe task (attentional bias retraining). A button feature to toggle between either task was also coded into the smartphone app.

### Task Design

A standard visual probe task [26] was used to measure attentional bias. The task comprised 100 trials, with each trial having a smoking-related image and a correspondingly matched neutral image. In each trial, participants were shown a fixation cross for 500 milliseconds before both images were displayed for 500 milliseconds. Following that, an asterisk replaced one of the images. In half of the trials, neutral images were replaced. Participants responded by clicking on the button at the position of the asterisk. The next trial would begin once a response had been recorded, or after 2000 milliseconds had passed, whichever came first.

In the assessment task, participants were presented with a fixation cross centered on the screen. Following the disappearance of the fixation cross, they were presented with a pair of stimulus images, with one image being related to smoking cues and the other being a neutral image but matched in terms of color and complexity. When these images disappeared, the participants were presented with a probe, and they had to register the position of the probe (left or right side of the screen) by pressing on the left or right on-screen buttons. In the assessment task, half of the trials involved the pairing of the probe with the smoking-related stimuli, and the other half involved pairing the probe with neutral stimuli. The modified visual probe task (attentional bias retraining) used the same task parameters as the standard visual probe task, the only difference being that the dot probe was consistently paired with the neutral stimuli to affect a shift in the attentional process.

The attentional bias score was tabulated as the median reaction time for trials in which the dot probe replaced the neutral image minus the median reaction time for trials in which the dot probe replaced the smoking image. A positive attentional bias score indicated a faster response to probes replacing smoking images than to probes replacing neutral images, suggesting attentional bias to smoking images. A negative attentional bias score indicated a faster response to probes replacing neutral images than to probes replacing smoking images, suggesting an attentional shift away from smoking images.

### Study Design

#### Approval and Consent

Ethics approval (approval number H7616) was obtained from the human research ethics committee at the institution that hosted the study. Before the start of the study, participants were provided with an information sheet and an informed consent form. They were notified that their participation was voluntary and that they had the right to withdraw at any time without explanation or prejudice. Participants were randomized into two groups: 5 in the control group and 5 in the training group. Each participant completed a demographic questionnaire. Subsequently, they were given a link to download and install the novel smartphone app on their Android phone. Each participant was allocated a personal log-in code and password to access the app.

#### Phase 1: Quantitative Trial

Similar to previous research [41], phase 1 involved the quantitative pilot trial, whereby 10 participants were randomly assigned to two groups—a training group (group 1) and a control group (group 2). Participants in the training group were informed about their allocation to group 1 and were asked to access the novel smartphone app in weeks 1, 2, and 3 of the study. Participants in the control group were informed about their allocation to group 2 and were asked to access the smartphone app in weeks 1 and 3 of the study. In week 1, participants from both groups attempted the standard visual probe task. They were instructed to respond quickly and accurately to the location of the dot probe that replaced either the smoking-related or the neutral stimulus. In week 2, the participants from group 1 attempted the modified visual probe task. Participants were instructed to complete this task in week 2 across 3 sessions. In week 3, participants from both groups attempted the standard visual probe task.

In week 1, participants in both groups (groups 1 and 2) were asked to complete a brief demographic questionnaire and rate their smoking frequency and subjective craving. Participants in both groups were also reminded to rate their smoking frequency and subjective craving in week 3.

#### Phase 2: Qualitative Process

Similar to previous research [41], phase 2 involved the qualitative process, whereby all 10 participants from both the training group (P1, P2, P3, P4, and P5) and control group (P6, P7, P8, P9, and P10) were invited to a semistructured interview after week 3. All participants were asked to describe their views and experience of the novel app, discuss specific areas that could be problematic, and provide their recommendations. Before the interviews, an interview guide was developed to ensure that all participants received similar prompts from the interviewer and to facilitate consistency in eliciting data. The interviews were audio-recorded and transcribed verbatim.

### Data Analysis

For the qualitative analysis, as in the study by Davies et al [45], 2 researchers coded the transcripts independently and manually using a thematic matrix technique within the framework of inductive coding. Upon completion of the coding, analyses were compared, any discrepancies were considered, and a consensus was reached. Similar to a previous study conducted on a novel smartphone app [41], thematic analysis was used to analyze the data from the interviews. The following six phases of analysis were employed: familiarization with the data, generating initial codes, searching for themes, reviewing themes, defining and naming themes, and final reporting using selected extracts. Before progressing into the coding phase, the data from the interviews were read and reread several times, resulting in data immersion. The codes identified the most basic features of raw data relevant to the research question. The coding process involved a constant backward and forward movement within the dataset to analyze the extracts that had been initially identified. Rigorous notetaking was undertaken in the coding process, and coding schemes were identified through the annotation of ideas. Using a thematic map, the codes were then sorted according to their similarities into identified themes. Additional reviews were conducted to ensure that no codes were omitted. All initial codes relevant to the research questions were incorporated into a theme.

A theme was composed of coded data grouped together according to their similarity [46,47]. In the context of our study, a theme would have to be relevant to our research topic of exploring participants’ perceptions and experiences of using the novel smartphone app. After the candidate themes were identified, a process of refinement of this collection of themes was undertaken. Per expert guidelines [46], this process involved reviewing the collated codes and themes, looking for internal and external homogeneity, and checking for coherence and accuracy of themes in relation to the data set as a whole. Various links and distinctions between the themes were drawn. The purpose of this refinement process was to ensure that the themes were all broadly related to one another in relation to the research question, while being distinct enough to be conceptualized on their own. The naming process of the themes involved a clear definition with a detailed analysis. Specific examples of each theme were selected to illustrate the different elements of each theme. These will be detailed in the *Results* section.

To enhance the credibility of the qualitative study and trustworthiness of the data, a few strategies were used. To accomplish investigator triangulation, 2 researchers independently analyzed the data. The researchers discussed and finalized the coded data from the interviews with the research supervisor to ensure that the themes best represented the participants’ perspectives. This also helped uncover any researcher bias that may have affected the integrity of the data. Both quantitative and qualitative results have been presented in the *Results* section, and their integration has been discussed in the *Discussion* section.

## Results

### Quantitative Analysis

We performed all quantitative analyses using SPSS (version 22.0; IBM, Inc). For the visual probe tasks, those with reaction times <200 milliseconds and incorrect responses were excluded. We analyzed the median reaction times to reduce the influence of the outliers. Quantitative data were analyzed using a series of Mann–Whitney *U* tests to examine the difference between the 2 groups (training vs control) with scores on attentional bias, subjective craving, and smoking frequency as the dependent variables over 2 time points (weeks 1 and 3). Descriptive statistics such as the means and SDs for attentional bias, subjective craving, and smoking frequency are presented in Table 1.

**Table 1:** Means and SDs of attentional bias scores, subjective craving, and smoking frequency for the study participants (n=10).

Time point		Training group (n=5) score, mean (SD)	Control (n=5) score, mean (SD)
**Attentional bias**			
	Week 1	−23.93 (80.26)	91.00 (161.40)
	Week 3	−27.24 (57.20)	2.89 (30.67)
**Subjective craving**			
	Week 1	5.00 (21.60)	5.00 (1.63)
	Week 3	4.00 (0.82)	4.70 (1.71)
**Smoking frequency**			
	Week 1	18.75 (9.43)	20.00 (11.43)
	Week 3	15.25 (9.84)	18.75 (11.12)

Participants in the training group were hypothesized to have decreased levels of attentional bias, subjective craving, and smoking frequency, as compared with participants in the control group. We conducted Mann–Whitney *U* tests to examine differences between the 2 groups (training vs control) with scores on attentional bias, craving, and smoking frequency as the dependent variables over 2 time points (weeks 1 and 3).

In week 1, the attentional bias score of those in the training group (mean rank 3.75) was not significantly different from the scores of those in the control group (mean rank 5.25; *U*=3.00; *z*=−1.44; *P*=.20, 2-tailed). Similarly, subjective craving of participants in the training group (mean rank 4.38) was not significantly different from that of participants in the control group (mean rank 4.63; *U*=7.50; *z*=−0.15; *P*=.89, 2-tailed). Similarly, the smoking frequency of those in the training group (mean rank 4.50) was not significantly different from that in the control group (mean rank 4.50; *U*=8.00; *z*=0.00; *P*=.99, 2-tailed).

In week 3, the attentional bias score of those in the training group (mean rank 4.00) was not significantly different from that in the control group (mean rank 5.00; *U*=6.00; *z*=−0.58; *P*=.69, 2-tailed). Similarly, the subjective craving of participants in the training group (mean rank 4.00) was not significantly different from that of participants in the control group (mean rank 5.00; *U*=6.00; *z*=−0.60; *P*=.69, 2-tailed). Similarly, the smoking frequency of those in the training group (mean rank 4.00) was not significantly different from that in the control group (mean rank 5.00; *U*=6.00; *z*=0.59; *P*=.69, 2-tailed).

### Qualitative Analysis

#### Overview

For the qualitative analysis, the thematic analysis process that was applied to the textual data elicited key concepts that were evident in the data. The codes were categorized into the following 5 themes: ease, helpfulness, unhelpful aspects, barriers, and refinement. The following subsections include the extracts that capture the essence of the respective theme without unnecessary complexity.

#### Ease

The theme of ease captured the participants’ perspectives on ease and simplicity, which enhanced the users’ experience. There was consensus among all participants regarding the ease of use of the app:

It has a straightforward design and simple interface; The simplicity is good.

P1

It’s rather intuitive and easy to use. I like that it’s very easy to use.

P2

Simple design.

P5

It’s just simple.

P6

Simple game. Very easy to use.

P8

Pretty easy to use. Straightforward.

P9

#### Helpfulness

The theme of helpfulness captured the perspectives expressed by participants in the training group. When describing their views and experience of the benefits, 60% (3/5) of participants from the training group who undertook attentional bias retraining noticed that they smoked less, and 40% (2/5) of participants perceived that their craving had decreased:

After using the app I seem to smoke less.

P1

Very helpful. After doing the app I really smoked a lot less. And I like it helped me reduce smoking.

P3

Quite helpful. I smoke a bit less now.

P5

#### Unhelpful Aspects

The theme of unhelpful aspects captured the perspectives expressed by participants in the control group on aspects that were unhelpful. When describing their views on the app, 40% (2/5) of participants from the control group who did not undertake attentional bias retraining felt that the app was not helpful and 60% (3/5) of them noticed no difference in their craving or frequency of smoking:

Same, I still smoke one pack every day.

P6

No effect, I still smoke the same.

P8

Don’t think it was helpful. Not even sure how it’s supposed to help with smokers.

P9

I don’t think it was helpful. No, still one pack every day.

P10

#### Barriers

The theme of barriers captured the perspectives that barriers included the amount of time needed to use the app, the lack of motivation, and forgetting to use the app because of busy personal schedules:

Time-consuming.

P1

Time-consuming...busy until forget [to] do...

P2

Quite a lot (of) time...3 times a week.

P4

Too long...forget [I] must do so many times.

P5

Take a lot of time.

P7

A little boring.

P9

Take up time.

P10

#### Refinement

The theme of refinement encapsulated the perspectives on features that could enhance the users’ experience, including details such as reminders, intervals, reduction in the number of items, task duration and frequency (once a week), lengthening the duration of each picture by 1 to 2 extra seconds, removing the password or having automatic log-in on subsequent use, and clicking on the picture instead of the button.

#### Other Subthemes

Pertaining to the subtheme of item reduction, 3 comments were made (P1, P7, and P5). On the subtheme of slower picture transition, 3 comments were made (P3, P6, and P7). With regard to the subtheme on the reminder, 2 comments were made (P5 and P8), and the subtheme on pause and break had 2 comments (P9 and P10). On the subtheme of password removal, auto log-in for subsequent use, and pressing on the photo instead of the green button, one comment was made by P2, P7, and P4:

Less items. About five minutes is good.

P1

So many photos.

P7

Half the number of pictures [is] best...do once a week [is] better.

P5

Password is unnecessary.

P2

Auto-login for subsequent use.

P7

The time from one picture to next [is] too fast...1 extra second each time will be good.

P3

The pictures change too fast...1 to 2 [extra] seconds will be good.

P6

Make it so the change [is] not too fast.

P7

Just let us press on the photos [is] less confusing. Green button [is] unnecessary.

P4

I need reminder to do.

P5

Alarm to remind us to do.

P8

Interval for break amidst the task.

P9

Give the option to pause and break.

P10

## Discussion

### Main Findings

This pilot study aimed to explore the use of a novel smartphone app in attentional bias retraining in smokers by using a mixed methods design. The main findings of the quantitative phase did not support the hypothesis that participants in the training group who undertook attentional bias retraining via the smartphone app would have decreased attentional bias, smoking craving, and smoking frequency, as compared with participants in the control group. The findings were not consistent with previous research, for example, by Kerst et al [38], who had found that attentional bias retraining delivered over a week led to a decrease in attentional bias and subjective craving for smokers in the training group. Although the outcome from the quantitative phase did not support the hypothesis, the outcome of the qualitative phase provided some preliminary evidence that participants in the attentional bias retraining group expressed no report of unhelpfulness.

### Strengths and Limitations

The perceived benefits from those who undertook attentional bias retraining included a decrease in craving and smoking frequency. However, the limitations should also be acknowledged. The major limitation of this study is the small sample size. Future studies should use a larger sample size informed by a power analysis. Nevertheless, findings from this pilot study can be used to inform the refinement of the novel app, which can then be used in a larger scale project, involving collaborations with community and industry partners at the local level and within the region.

There might be extraneous variables inherent in the sample at baseline. As cultural variations can contribute to vulnerabilities and resilience in a range of health issues [40], which might include nicotine dependence, future research should endeavor to collate further details on culture and incorporate a questionnaire such as the Fagerström Test for Nicotine Dependence [48] to assess nicotine dependence at baseline.

Another limitation was regarding the choice of stimulus materials, which should be informed by findings from a preliminary focus group. The design of the questions in the focus group can be conceptualized to examine the questions posed by the research by Woud et al [44]. The purpose of the focus group can include examining sensitivity to the variety of smoking-related images, the effect of the duration of exposure, the order of materials, and the pairing of images. The outcome can then be used to inform the choice of neutral images, as well as the final design of the stimulus materials.

Further improvements can be made to the research design. In our study, the participants were randomly assigned to the experimental and control groups. The design can be improved if both groups matched in their key characteristics. In addition, the scales used in this study (ie, the visual analog and smoking frequency scales) are both based on self-reports, and thus, subject to recall bias and social desirability bias. There could also be other extraneous factors, as described in the subsequent sections.

First, our study was the first of its kind to deliver attentional bias retraining for smokers via a novel smartphone app. Unlike the study conducted by Kerst et al [38], which provided participants with a PDA and previous laboratory studies, there might be possible distractions from personal mobile notifications while our participants undertook attentional bias retraining on their mobile phones. This might contribute to loss of focus, which could lead to a decreased possibility of attentional bias change [49]. Second, unlike our study, which comprised 3 sessions of attentional bias retraining over a week, Kerst et al [38] delivered attentional bias retraining 3 times daily over a week. The lower training frequency in our study might have been insufficient to produce significant changes in attentional bias. This suggests that attentional bias may be relatively less malleable to change unless there was a higher load of training involved. Hence, future studies can adhere to multiple training sessions daily for at least a week, as previous research has demonstrated that only those studies that used multiple trainings daily for a week or more had robust results [38,50].

Future studies can include features to block incoming calls and notifications to minimize distractions on mobile phones while participants are engaged in the training. This can maximize their focus and minimize extraneous factors that might diminish the effect of the training [49]. The study can also be replicated with increased training frequency and provision of incentives for completion, which might enhance the motivation to commit to the multiple sessions of daily training required. Using hospital-based samples or recruiting from smoking cessation programs in the community might also enhance engagement and minimize attrition rates.

### Themes Identified

The qualitative study aimed to explore the participants’ perceptions and experiences of the novel smartphone app. The following 5 themes emerged from their responses: ease, helpfulness, unhelpful aspects, barriers, and refinement. The theme of ease was consistent with previous research that described the benefits of health-related mobile apps. Our finding was consistent with a recent study [51] that explored the perspectives of participants on the use of mobile health apps. Similarly, the app was viewed favorably with regard to its ease of use and convenience. Ease of use is often correlated with sustained use [52], and coupled with the convenience of mobile devices [53], this could maximize treatment adherence to attentional bias retraining, given the high frequency of attentional bias retraining required for robust outcomes.

The themes of ease of use and helpfulness were consistent with previous research [54] and aligned with effort expectancy, which is a construct based on the idea that there is a relationship between ease of use and the rewards from the effort [55]. Previous studies found that the harder the participants perceived it was to operate a mobile app, the less they used it to reap its benefits [56]. Therefore, the ease of use and the convenience of attentional bias retraining in the novel app can help enhance its use among smokers.

The theme of helpfulness was consistent with a recent qualitative study [54], which explored the benefits of a mental health mobile app. Participants perceived the app to be helpful in their recovery process, as well as a good complement to traditional follow-up methods for posttrauma symptoms. In contrast, participants in our control group did not undertake attentional bias retraining and subsequently perceived the aspect of the app they received as being unhelpful, thus providing preliminary evidence on perspectives that demarcated the training and control groups.

The theme of barriers perceived by the participants was consistent with that of a recent study [56]. Similar to Peng et al [56], where participants perceived that a lack of discipline and time commitment contributed to the barrier to their smartphone app use, the smokers in our study found that the required time commitment was a barrier for their consistent use of the app. Future studies should engage in comprehensive usability testing to determine ideal scheduling while maintaining robust outcomes. Further in-depth interviews can uncover the underlying factors that might enhance the commitment to sustained and consistent engagement with the app. In future research, the use of in-built personalized reminder features can also help facilitate the participants to use the mobile app at a time that is most convenient to them. The theme of refinement is aligned with the study by Anderson et al [51], where suggestions were made to improve their novel mobile health apps. Specifically, the suggestions included fine-tuning of certain features. Further enhancement of our app could include an auto log-in function, scheduling of intervals, and a more intuitive button response within the app. In addition, future studies can consider gamification of such applications to increase engagement and sustain motivation [57] or incorporate rewards to encourage use and investigate their pros and cons. Owing to time and resource constraints, follow-up was not possible in this study. Ideally, it would be best if the themes elicited could be presented to the participants for checking and further refinement. In hindsight, it would be optimal to design the research question for phase 1 by incorporating the barriers and operationalize it by investigating the specific duration of time that participants spent each week on the app.

### Conclusions

This study provides findings from both quantitative and qualitative research methods. Although the quantitative outcome did not support the hypothesis, the qualitative outcome provided preliminary support for the benefits of the novel smartphone app. The identified themes of ease, helpfulness (pertaining to those who undertook training), unhelpful aspects (pertaining to those who did not undertake training), barriers, and refinement were consistent with previous research. This study provides preliminary evidence to support some benefits of the novel smartphone app for smokers. The qualitative findings can be used to refine the app for use in larger scale studies to further explore the effects of the novel mobile app, which might lead to the future possibility of its use in smoking cessation programs. However, the overall findings of this study should be interpreted with caution, as its small sample size is a limitation. Nonetheless, the qualitative results were useful in understanding the perspectives and experiences of participants who used the novel app and can further inform future research on this pertinent topic.
